# Study on the Mechanical and Mesoscopic Properties of Rockfill Under Various Confining Pressures

**DOI:** 10.3390/ma18061316

**Published:** 2025-03-17

**Authors:** Bin Ou, Haoquan Chi, Zixuan Wang, Haoyu Qiu, Jiahao Li, Yanming Feng, Shuyan Fu

**Affiliations:** 1 College of Water Conservancy, Yunnan Agricultural University, Kunming 650201, China; oubin418@126.com (B.O.); wangzixuan010124@163.com (Z.W.); 18987275348@163.com (H.Q.); 13509761797@163.com (J.L.); 2 Yunnan Key Laboratory of Water Conservancy and Hydropower Engineering Safety, Kunming 650051, China; 18208891690@163.com; 3 National Key Laboratory of Water Disaster Prevention, Hohai University, Nanjing 210098, China

**Keywords:** granular materials, stress-strain behavior, discrete element method, PFC^2D^ modeling, triaxial shear test

## Abstract

To investigate the mechanical response characteristics of damming rockfill materials under different confining pressure conditions, this study integrates laboratory triaxial compression tests and PFC^2D^ numerical simulations to systematically analyze their deformation evolution and failure mechanisms from both macroscopic and microscopic perspectives. Laboratory triaxial test results demonstrate that as the confining pressure increases, the peak deviatoric stress rises significantly, with the shear strength of specimens increasing from 769.43 kPa to 2140.98 kPa. Under low confining pressure, rockfill exhibits pronounced dilative behavior, whereas at high confining pressure, it transitions to contractive behavior. Additionally, particle breakage intensifies with increasing confinement, with the breakage rate rising from 4.25% to 8.33%. This particle fragmentation alters the granular skeleton structure, thereby affecting the overall mechanical properties and leading to a reduction in shear strength. Numerical simulations further reveal the micromechanical mechanisms governing rockfill behavior. The simulation results show a shear strength increase from 572.39 kPa to 2059.26 kPa, exhibiting a trend consistent with experimental findings. The shear failure mode manifests as a characteristic “X-shaped” shear band distribution, while at high confining pressures, shear fracture propagation is effectively inhibited, enhancing the overall structural stability. Furthermore, increasing confining pressure promotes denser interparticle contacts, with contact numbers increasing from 16,140 to 18,932 and the maximum contact force rising from 12.19 kN to 59.83 kN. The quantity and frequency of both strong and weak force chains also increase significantly, further influencing the mechanical response of the material. These findings provide deeper insights into the mechanical behavior of rockfill materials under varying confining pressures and offer theoretical guidance and engineering references for dam stability assessment and construction optimization.

## 1. Introduction

The strength, deformation characteristics, and particle breakage characteristics of rockfill materials are important mechanical characteristics that constitute the theoretical basis of engineering design, such as earth-rock dams, high-fill slopes, railways, and highways [1]. The complex soil structure of rockfill materials is affected by many factors, including stress state, loading path, particle shape, and size, etc. In practical engineering applications, due to differences in construction technology and geological conditions, the stress paths of rockfill materials show diversity [2,3]. Therefore, it is of great theoretical and practical significance for the design, construction, and long-term stability of earth-rock dams to study the macroscopic and mesoscopic mechanical behaviors of rockfill materials under different confining pressures and reveal their deformation and failure mechanisms.

Scholars at home and abroad have conducted a lot of research on the strength, deformation characteristics, and particle breakage of rockfill materials, and achieved fruitful results [4,5]. In the prediction of the strength and deformation characteristics of rockfill materials, Sadeghian et al. [6] studied the mechanical behavior of shell conglomerate rockfill materials of Masjed Soleyman dam through point load, Brazilian splitting, consolidation instruments, and direct shear tests. The results show that the strength of the material is affected by water content and particle size, and the degree of particle breakage is more significant in direct shear testing, which is suitable for predicting the shear deformation behavior of the dam. The ratio of particle breakage to strain is not affected by stress and moisture content, but is significantly affected by gradation and density. Wet compaction can effectively reduce the problem of dam settlement and saturated settlement. Yang et al. [7] established a CNN model to predict the permeability coefficient of rockfill materials by using particle parameters and void ratio. The results show that the model is superior to the BP model and is suitable for rapid estimation in engineering. Li et al. [8] predicted the shear strength of rockfill materials by the SVR model optimized by LogSAO. The results show that the predicted value of the model is highly consistent with the measured value and has the best performance. On the failure and deformation behavior of rockfill materials, Du et al. [9] and Wang et al. [10] studied the failure characteristics of clay under different water conditions by triaxial compression tests. Wang et al. [11] used the discrete element method to study the deformation and failure of a soil-rock mixture and revealed the phenomenon of flow around the rock block. Hu et al. [12] studied the failure mode of an artificial frozen soil-rock mixture and pointed out that under compression, it is mainly manifested as expansion deformation and short tensile cracks, and indirect tension produces tortuous tensile cracks. For the study of particle breakage, Guo et al. [13] and Luo et al. [14] established a particle breakage rate model through triaxial tests and proposed relevant mathematical expressions and determination methods of model parameters. Zhou [15] studied the creep deformation of delayed particle failure in rockfill materials and proposed the important influence of long-term strength and maximum contact force on particle failure. The cyclic loading behavior of rockfill materials has also been studied in depth. Yan et al. [16] studied the cyclic loading behavior of rockfill materials through a particle strength statistical model and DEM simulation. It was found that the large particle size samples were more broken and the structure was denser, and Young’s modulus increased significantly with the loading frequency. Zhou et al. [17] studied the creep deformation of delayed particle failure in rockfill materials and proposed the important influence of long-term strength and maximum contact force on particle failure. In terms of strengthening the performance of rockfill materials, Yu et al. [18] significantly improved the tensile properties of slag cement rockfill materials by adding carbon nanotubes (CNTs), which improved the tensile strength and delayed microcrack propagation. On the scale effect of rockfill materials, Han et al. [19] simulated rockfill materials with different particle sizes by mixing method and studied the scale effect of particle size on rockfill materials, proposing that the increase in particle size will reduce the peak deviator stress and phase transition point stress ratio while increasing the cohesion. Zhao et al. [20] proposed a gradation evolution model and an elastic-plastic constitutive model through a single particle crushing test, which can predict the stress-strain behavior and gradation evolution of rockfill under shear loading, and verify the linear correlation of the crushing index. In the numerical simulation of rockfill materials, the PFC particle flow method has become the core technology for analyzing the meso-mechanical properties and has been widely used in the study of triaxial compression, shear characteristics, and crack evolution. A number of studies have shown that the PFC model can effectively reproduce the mechanical behavior and meso-evolution characteristics of rockfill materials [21,22]. First, PFC has shown great ability in studying the influence of particle distribution and mechanical properties. Based on the CSM and PFC models, Yang et al. [23] studied the rock-breaking law of super-hard rock shields in Fuzhou Metro. The results show that the cutter force changes with the position and quantity, the cracks gradually penetrate, and the mechanical properties meet the engineering requirements, which provides a reference for shield construction. Han et al. [24] studied the effect of fractal dimension on particle distribution and mechanical properties and found that the increase in fractal dimension can improve the strength and tightness. In the study of interface mechanical properties, the PFC model shows high precision. Similarly, Jing et al. [25] used PFC^2D^ to study the crack propagation law of fractured rock mass under uniaxial compression. It was found that the increase in fracture dip angle would reduce the peak stress and increase the sensitivity of crack propagation stress and Poisson’s ratio, which provided a theoretical basis for the stability control of underground rock mass. In the study of interface mechanical properties, the PFC model shows high precision. Wan et al. [26] simulated the shear characteristics of the frozen soil–concrete interface by PFC, accurately reproducing the formation of shear bands and the microscopic response of particles, and the regression equation further predicted the shear behavior of the interface under different temperatures and stresses. Chen et al. [27] studied the influence of different normal stresses and microscopic characteristics of joints on single-bolted rock joints by PFC numerical calculation. It was found that bolts enhanced the shear strength, and the increase in tangential stiffness and friction coefficient of joints increased the shear strength, while the increase in joint thickness reduced the shear strength and reduced the number of cracks. In addition, PFC is also used to simulate the mechanical behavior of complex rock mass and coarse-grained soil. Li et al. [28] analyzed the acoustic emission (AE) response and damage evolution of silty clay under uniaxial load through experiments and PFC simulation, revealed the five stages of AE response, and proposed a damage model that accurately reflected the stress-strain dynamics. The PFC^3D^—FLAC^3D^ coupling algorithm proposed by Bai et al. [29] successfully simulated the rock mass disturbance in tunnel excavation, optimized the energy reflection problem, and effectively characterized the stress distribution and crack propagation process. Finally, PFC is outstanding in the study of coarse-grained soil. He et al. [30] used PFC^3D^ to simulate the triaxial compression test of coarse-grained soil, revealing the hysteresis of bond failure between soil particles and its correlation with the macroscopic stress-strain curve. Xing et al. [31] further analyzed the influence of coarse grain content on the elastic modulus and shear strength of coarse-grained soil, while Zhang et al. [32] simulated the mesoscopic changes of coarse-grained soil under triaxial compression by PFC–FLAC coupling, and found that its deformation and failure were mainly caused by particle movement, pore change, and the evolution of particle structure characteristics.

Although the existing research has made many contributions to the structural characteristics and mechanical response characteristics of rockfill materials, there are still some shortcomings in the existing research due to the complex structure of rockfill materials, the diversity of test conditions, and the possible errors in data processing. Therefore, it is particularly important to explore the comparative relationship between the macroscopic and mesoscopic mechanical properties of rockfill materials. In this study, the dynamic and static triaxial test machine was used to carry out indoor experiments, and combined with PFC numerical simulation software, the mechanical response characteristics of rockfill materials in the process of shear failure were analyzed from the microscopic point of view. Through the comparative analysis of the macroscopic and mesoscopic changes of rockfill materials, this study reveals the mechanical response characteristics of rockfill materials and provides scientific guidance for further improving the application safety and construction level of rockfill materials in engineering.

## 2. Conventional Triaxial Compression Test

### 2.1. Test Materials

In the study of embankment engineering, rockfill materials typically consist of coarse particles (d > 5 mm) and fine particles (d < 5 mm). The coarse particles form a skeletal structure, providing the primary load-bearing capacity, while the fine particles fill the voids within the skeleton, enhancing overall compactness and shear strength, and mitigating settlement deformation. Therefore, the grain composition is a critical factor influencing the engineering properties of rockfill materials. In practical engineering applications, a comprehensive analysis of the grain size distribution is essential for optimizing design and construction.

The test materials used in this study were sourced from the rockfill materials of an embankment slope in a dam located in Yunnan, China. The material primarily consists of gravel, boulders, and a soil-rock mixture containing sand and clay, with lithology dominated by highly weathered and moderately weathered sandstone and limestone. Due to weathering processes, the rock becomes loose and readily fractures into suitable particle sizes, making it an ideal rockfill material. The material exhibits a specific gravity of 2.8, with a natural moisture content of 3.45%, a plastic limit moisture content of 4.08%, and a liquid limit moisture content of 11.92%.

**Figure 1:**
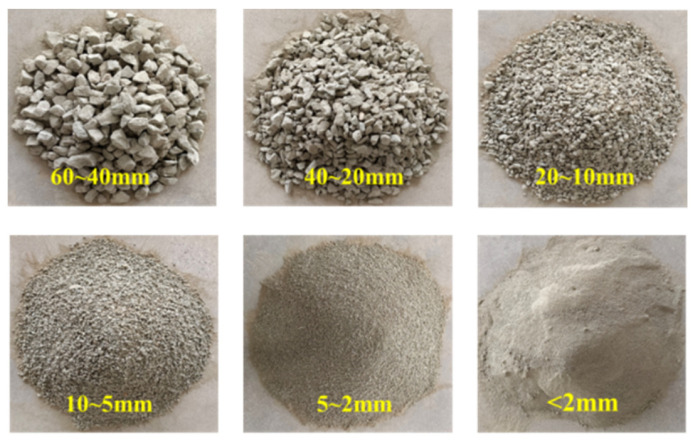
Characteristics of rockfill particle group.

The rockfill particles exhibit relatively regular morphology and rough surfaces, which enhance interparticle friction and interlocking, thereby improving the overall stability of the dam structure. Additionally, the mountainous terrain and significant fluvial erosion in Yunnan result in a wide range of grain size distributions, from large boulders to fine sand, enabling the material to meet the requirements of various dam designs. Notably, these rock types possess high strength and stability, and under high confining pressure, they exhibit excellent shear strength, contributing to improved dam stability and load-bearing capacity. The grain size characteristics of the rockfill material are illustrated in Figure 1.

To accurately determine the grain size distribution of rockfill materials, appropriate testing methods should be selected based on particle size. Fine particles (d < 5 mm) are measured using the sedimentation analysis method, while particles within the sieving range (5 mm < d < 60 mm) are analyzed through sieve analysis. For oversized rock blocks (d > 60 mm), the direct measurement method is used for statistical assessment. In this study, sieve analysis was employed to determine the particle size distribution of the specimens, with sieve apertures of 60 mm, 40 mm, 20 mm, 10 mm, 5 mm, and 2 mm, ensuring coverage of the primary grain size fractions.

Due to the large variation in rockfill particle sizes and the limitations of laboratory equipment in specimen preparation, particle size adjustment was necessary. According to the Standard for Geotechnical Testing Methods (GB/T 50123-2019) [33], the maximum particle size of a specimen should not exceed 1/6 to 1/5 of its minimum dimension. In this study, the standard specimen diameter is 300 mm, corresponding to a maximum allowable particle size of 60 mm.

**Figure 2:**
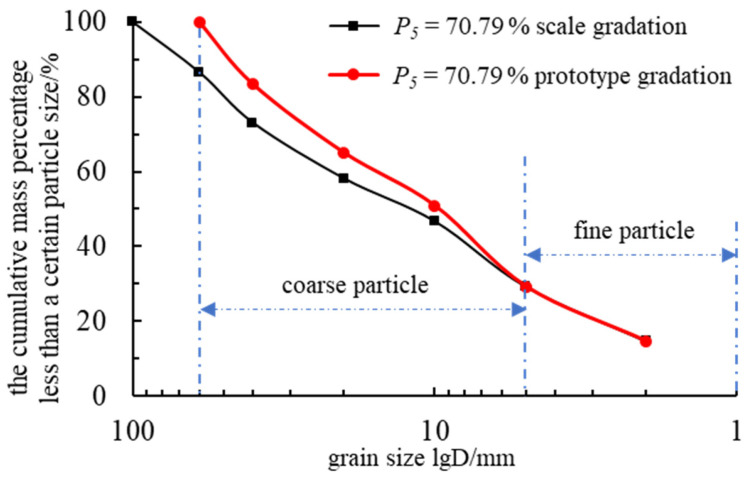
Accumulation curve of rockfill particle size distribution.

A significant proportion of the test material consisted of oversized particles (d > 60 mm), and directly omitting them could lead to distortions in the gradation characteristics. Therefore, an equivalent replacement method was applied, in which smaller-sized particles were substituted in an equivalent proportion for oversized particles [34]. This approach ensures compliance with the particle size requirements of the testing apparatus while preserving the original gradation characteristics of the rockfill as much as possible, thereby minimizing potential bias in experimental results. The adjusted grain size distribution is presented in Table 1, and the cumulative particle size distribution curve is shown in Figure 2.

### 2.2. Test Equipment and Scheme

**Figure 3:**
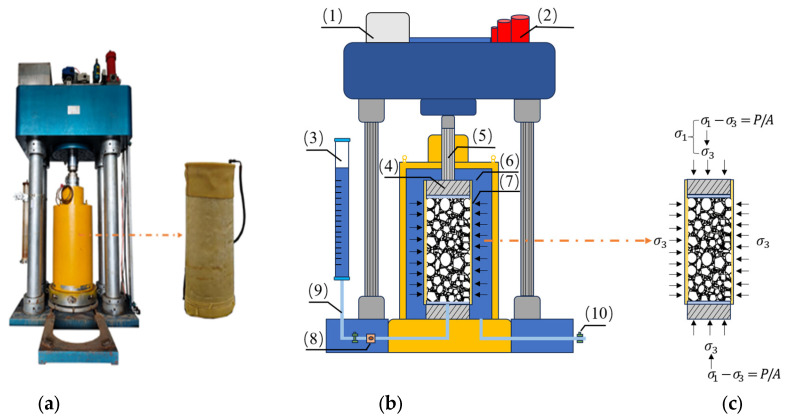
(**a**) Static and dynamic triaxial testing machine; (**b**) three-axis mechanism manufacturing; (**c**) the stress state of the test soil sample: (1) water tank; (2) energy storage tank; (3) volume change tube; (4) permeable plate; (5) force transmission column; (6) pressure chamber; (7) latex film; (8) sensor; (9) drainage pipe; (10) valves. DJSZ-150 large-scale dynamic and static triaxial testing machine for coarse-grained soil.

The triaxial compression test is a laboratory testing method used to determine the strength and deformation characteristics of soil. DJSZ-150 Large-Scale Dynamic and Static Triaxial Testing System (Chengdu Donghua Excellence Technology Co., Ltd., Chengdu, China) was utilized in this study. This large-scale triaxial compression apparatus is capable of preparing coarse-grained soil specimens with a diameter of 300 mm and a height of 600 mm, as shown in Figure 3. Depending on drainage conditions, triaxial tests can be conducted under unconsolidated undrained (UU), consolidated undrained (CU), or consolidated drained (CD) conditions, where the drainage valves remain closed throughout the entire test in the UU test, are open only during consolidation in the CU test, and remain open throughout both consolidation and shear in the CD test to allow full dissipation of pore water pressure. The selection of an appropriate test type should be based on actual soil conditions to accurately reflect the drainage behavior of the soil during shear.

The triaxial compression apparatus used in this study consists of several key components, including a water chamber, energy storage tank, volume change measurement device, porous plates, load transfer column, pressure chamber, latex membrane, sensors, drainage pipes, and valves. During testing, the specimen is encapsulated in a latex membrane and placed within a sealed pressure chamber. Confining pressure σ_3_ is applied by injecting pressurized fluid through a valve, while an axial deviatoric stress σ_1_ − σ_3_ = *P*/*A* (where *P* is the axial load and *A* is the cross-sectional area of the specimen) is applied through a piston rod, ensuring an axisymmetric stress state. Axial deformation is measured to calculate the corresponding axial strain ε_1_. By adjusting the confining pressure σ_3_ and deviatoric stress σ_1_−σ_3_, various stress path tests can be conducted. A conventional triaxial compression test (isotropic consolidation compression test) is performed under constant confining pressure σ_3_ while the axial stress and deviatoric stress σ_1_−σ_3_ gradually increase until specimen failure occurs.

This apparatus is capable of conducting both conventional triaxial tests and customized stress path tests. It accommodates specimens of Φ300 × 600 mm, with a maximum axial load capacity of 1500 kN and a maximum confining pressure of 3.0 MPa. The system is fully automated and capable of monitoring multiple test parameters, including axial stress, strain, confining pressure, pore pressure, specimen volume change, saturation water intake, and consolidation drainage volume. The technical specifications of the DJSZ-150 large-scale dynamic-static triaxial testing system for coarse-grained soils are presented in Table 2.

The test materials used in this study have a density of 2.32 g/cm³, a natural moisture content of 3.45%, and a designed coarse grain content of 70.79%. All the tests were conducted in strict accordance with the relevant provisions of the Standard for Soil Test Methods (GB/T 50213-2019) [35].

According to the scaled gradation data in Table 2, the dam rockfill material was reconstituted. The specific procedure is as follows. Five portions of dry soil were weighed, and the required amount of water was added and thoroughly mixed. The prepared soil samples were then compacted and filled into the specimen mold in five layers. To minimize the segregation of coarse and fine particles during filling, the surface of each compacted layer was roughened before placing the next layer. This process was repeated until the specimen mold was completely filled.

In the triaxial shear test, confining pressures were set at 100 kPa, 200 kPa, 300 kPa, 400 kPa, 500 kPa, and 600 kPa, with a total of six test groups. The test was conducted under consolidated drained (CD) conditions, with a loading rate of 1.35 mm/min, applying automated loading until the axial compression displacement of the specimen reached 15% of its original height, at which point shear failure was determined. The detailed test plan is presented in Table 3.

### 2.3. Establishment of PFC^2D^ Compression Model

PFC (Particle Flow Code), Itasca Consulting Group, Inc., Minneapolis, MN, USA, was used for the numerical simulation in this study. In order to ensure the accuracy and reliability of the simulation results, a script was written in the built-in Fish language of PFC^2D^, based on the size of the triaxial shear box (300 mm × 600 mm), to generate a model that meets the test requirements. In the process of model generation, according to the scale results of indoor triaxial test materials, the generated particles represent the particle size and distribution characteristics of the real samples, so as to simulate the behavior of rockfill materials more realistically. In order to more accurately simulate the compaction process of rockfill materials, the Layered Compaction Method is used to compact the particles layer by layer, one layer at a time, until the particles reach the target porosity of 0.195, and finally, a model containing 16,375 particles is formed [36].

**Figure 4:**
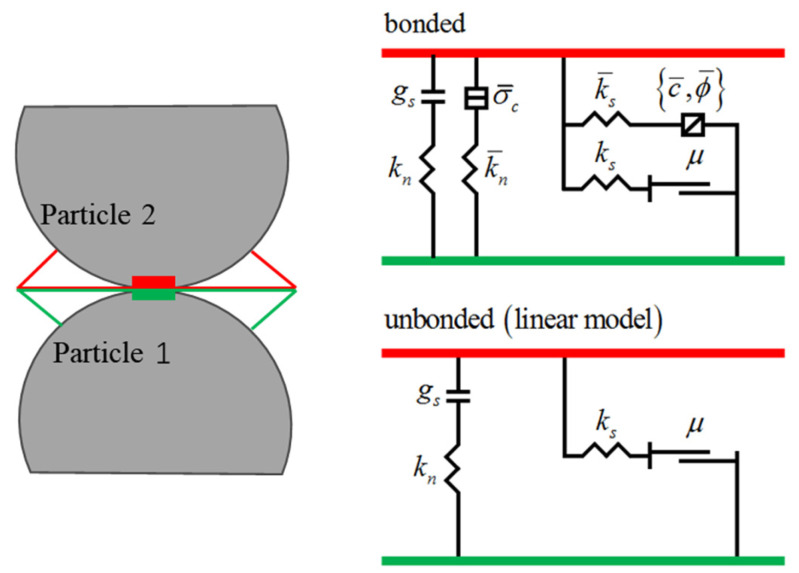
Schematic diagram of the parallel bonding model.

When simulating the shear failure process, in order to accurately reflect the mechanical behavior of rockfill materials in the shear failure process, the parallel bond model is selected for numerical simulation. In order to ensure that the simulation test can better fit the indoor test, the model uses rigid particles to ensure that there is no elastic deformation in the contact between particles. By setting the cohesive force between particles, the model can reasonably simulate the interaction and failure mechanism between particles, so as to effectively characterize the shear failure characteristics of rockfill materials. The parallel bond model is particularly suitable for describing rockfill materials with a granular structure. During the shear process, the interaction force and failure mode between particles will directly affect the macroscopic mechanical behavior of rockfill materials. Therefore, this model can more accurately simulate the various mechanical properties of the sample during the triaxial shear process [37]. The schematic diagram of the parallel bond model is shown in Figure 4.

It can be seen from Figure 4 that there are two kinds of contact interfaces in the parallel bond model. The first is the linear elastic contact interface, which is equivalent to the linear contact model; that is, it is composed of a vertical separator *g_s_*, normal stiffness *k_n_*, tangential stiffness *k_s_*, and friction coefficient *μ*. Due to the existence of a vertical separator, this kind of model can not bear tension but can bear friction. The second contact interface is a linear elastic bonding contact interface. Based on the linear elastic contact interface of this kind of contact interface, a cement ${\bar{\sigma }}_{c}$, a parallel bonding normal stiffness ${\bar{k}}_{n}$, a parallel bonding tangential stiffness ${\bar{k}}_{s}$, a parallel bonding cohesion $\bar{c}$, and a friction angle $\bar{\phi }$ are added. Due to the existence of the cement, it can withstand both tension and friction. When the soil is stressed, the parameters set on the parallel bond model can resist the action of force. As the external force continues, the soil particles will change their spatial position. If the external force exceeds the strength limit of the parallel bond model, the model will be destroyed, and the parallel bond model after destruction will be transformed into a linear model without a bond. At this time, the contact state between the soil particles is linear elastic contact.

In the PFC^2D^ shear numerical simulation test, the shear equation of the particles is usually based on the parallel bond model to describe the interaction between the particles. The model simulates the shear behavior of particles by setting the cohesive force between particles. The shear equation usually involves the calculation of the contact force between particles, especially the normal force and tangential force of the contact point. In the parallel bond model, the shear force of the particles can be described by the normal force, tangential force, and failure criterion (Coulomb friction criterion), namely

(1) $$F_{N}=k_{N}\cdot \delta$$

where *k_N_* is the normal stiffness and *δ* is the normal displacement between particles,

(2) $$F_{T}=\mu \cdot F_{N}$$

where *μ* is the friction coefficient between particles, and *F_N_* is the normal contact force, and

(3) τ=μ⋅σ

where *τ* is the tangential stress, *μ* is the friction coefficient, and *σ* is the normal stress.

**Figure 5:**
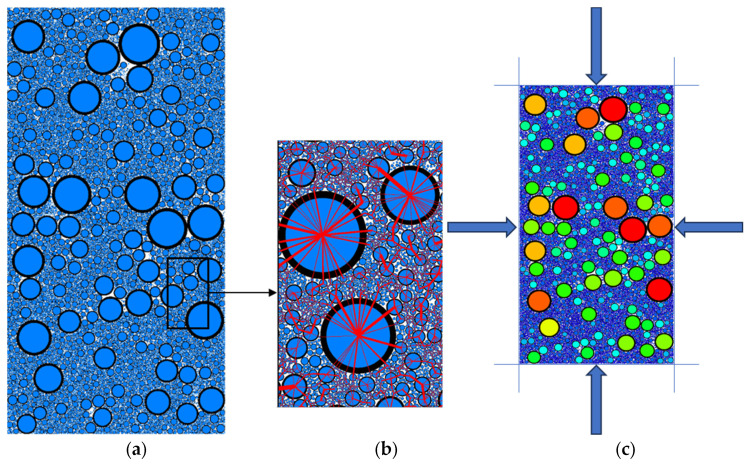
Model and model loading diagram: (**a**) model diagram; (**b**) parallel bond contact; (**c**) loading diagram.

The simulation results show that the model can effectively capture the shear failure process of rockfill, which is consistent with the actual behavior of rockfill, and verifies the rationality and effectiveness of the simulation method, as shown in Figure 5.

**Figure 6:**
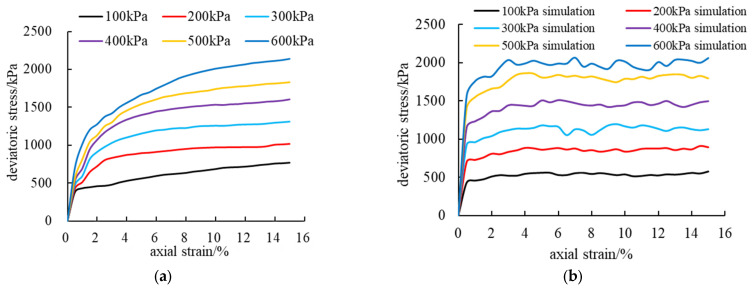
Comparison of test curve and simulation curve of coarse grain content *P*_5_= 70.79 rockfill material: (**a**) The test curve of rockfill material with coarse grain content *P*_5_ = 70.79. (**b**) The simulation curve of rockfill material with coarse grain content *P*_5_ = 70.79.

In order to realize the accurate calibration of PFC^2D^ numerical simulation parameters, this study uses the “trial and error method” to carry out a large number of trial calculations, and compares these trial results with laboratory test data to ensure the accuracy of the parameters [38]. According to the relevant macroscopic mechanical parameters obtained from the laboratory test, the researchers assigned the required microscopic mechanical parameters to the parallel bond model. In this process, firstly, the large indoor deviation stress–axial strain curve is used as the reference standard for simulation. Then, by comparing the PFC^2D^ numerical simulation results with the actual indoor direct shear test results, the meso-mechanical parameters between rockfill particles are gradually adjusted, and finally, the accurate calibration of the parameters is realized. The final results of parameter calibration are shown in Table 4, and the curve of the whole calibration process is shown in Figure 6. Through this method, the parameters in the model can be adjusted in detail, so that the simulation results are closer to the actual test data, and the accuracy and reliability of the numerical model in describing the shear behavior of rockfill materials are improved.

As shown in Figure 6, the deviation stress–axial strain curves of rockfill materials under different confining pressures (100 kPa to 600 kPa) were numerically simulated and compared with the indoor test results. The results show that the curve of numerical simulation is in good agreement with the actual measurement curve of the laboratory test, and both of them show obvious strain-hardening characteristics. This shows that with the increase in axial strain, the deviation stress continues to rise, and the material gradually shows stronger resistance to deformation.

Under the condition of confining pressure of 600 kPa, when the sample reaches the shear failure state of 15%, the shear strength obtained by numerical simulation is 2059.26 kPa, while the measured value of the indoor test is 2140.98 kPa. Although there are some differences between the two, the overall results are extremely close, indicating that the numerical simulation model can accurately reflect the actual mechanical properties of the material. However, in the process of shear failure, the numerical simulation curve has a certain degree of fluctuation, which is more obvious than the indoor test curve. The analysis shows that this fluctuation mainly comes from the following aspects. (1) The randomness of particle formation. In the PFC^2D^ model, the particles are assumed to be regular spherical particles, and the formation process has a certain randomness. This randomness leads to the local accumulation of coarse particles near the shear surface, which in turn, causes the composition and geometric arrangement of the particles inside the shear surface to be different from the laboratory test. This difference will directly affect the smoothness of the simulation results, which will lead to the fluctuation of the simulation curve. (2) The dislocation and occlusion between particles. In the simulation process, the dislocation and occlusion between coarse particles further aggravate the discontinuity of the stress curve. This phenomenon is more significant in numerical simulation because the arrangement and movement of particles have higher degrees of freedom, which causes fluctuations in the overall mechanical response. (3) The continuity of data recording. The data recording mechanism in the PFC^2D^ model may lack sufficient smoothing in some details. Particularly in the simulation of large deformation or failure processes, the sudden change in particle contact force and local stress field may lead to more significant nonlinear fluctuations in curve recording.

Despite the above shortcomings, in general, the deviation stress–axial strain curve obtained by the numerical simulation model based on the above calibration parameters is still highly correlated with the indoor test results. This shows that the PFC^2D^ model can reliably simulate the mechanical properties of rockfill materials under different confining pressures, especially in reflecting the strain-hardening characteristics and shear strength.

## 3. Comparative Analysis of Simulation Results and Indoor Results

### 3.1. Failure Mode Analysis of Laboratory Test and Numerical Simulation

**Figure 7:**
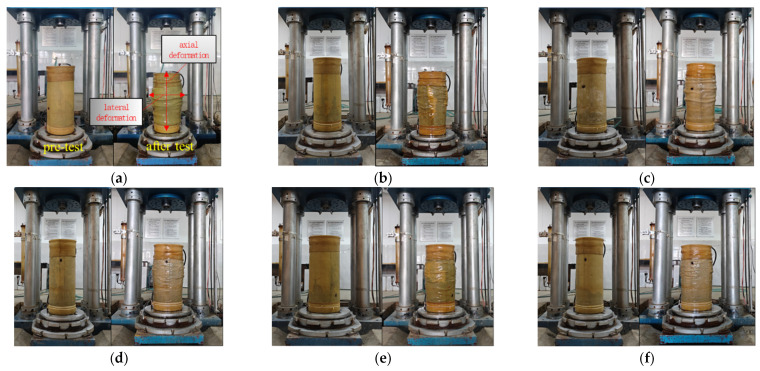
Failure characteristics of rockfill under triaxial compression test: (**a**) σ_3_ = 100 kPa. (**b**) σ_3_ = 200 kPa. (**c**) σ_3_ = 300 kPa. (**d**) σ_3_ = 400 kPa. (**e**) σ_3_ = 500 kPa. (**f**) σ_3_ = 600 kPa.

**Figure 8:**
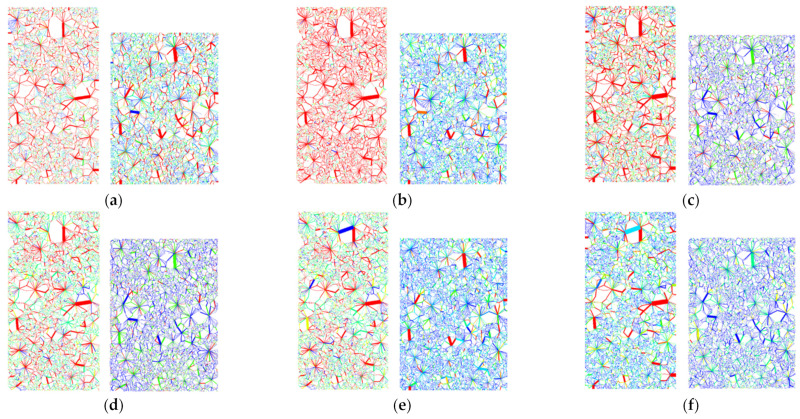
Numerical simulation of particle shear plane variation characteristics: (**a**) σ_3_ = 100 kPa. (**b**) σ_3_ = 200 kPa. (**c**) σ_3_ = 300 kPa. (**d**) σ_3_ = 400 kPa. (**e**) σ_3_ = 500 kPa. (**f**) σ_3_ = 600 kPa.

In the triaxial shear test, dam rockfill will not only produce shape deformation but also produce volume deformation. The volume change (volume expansion or volume contraction) caused by triaxial shear is collectively referred to as dilatancy. Dilatancy is an important factor in the deformation and failure of rockfill materials. The failure modes of the indoor triaxial compression test and PFC numerical simulation were compared and analyzed, as shown in Figure 7 and Figure 8.

The colors in the numerical simulation represent different contact force magnitudes: red indicates strong contact forces and primary force chains, green represents moderate contact forces and particle interactions, and blue corresponds to weak contact forces or minor particle movements. As shown in Figure 7, during the static loading process, the volume change of rockfill materials is mainly determined by axial deformation and lateral deformation and shows significant confining pressure dependence. With the change in confining pressure, the deformation characteristics of rockfill materials are obviously different: under low confining pressure (100 kPa and 200 kPa), the lateral expansion is particularly significant. This is because the lateral constraint is weak under low confining pressure, and the particles are mainly slippery. With the increase in axial stress, the slip of particles leads to an increase in lateral expansion, and the overall volume change is dominated by expansion. Under the condition of medium confining pressure (300 kPa and 400 kPa), the deformation characteristics of rockfill transition from lateral expansion to axial compression. With the increase in confining pressure, the contact between particles is enhanced, the constraint effect is improved, the particle slip is limited, and the volume change is mainly manifested as the compression in the axial direction. Under high confining pressure (500 kPa and 600 kPa), the lateral expansion phenomenon is obviously weakened, and the lateral deformation of rockfill tends to be stable. This is due to the significant enhancement of the contact force between particles under high confining pressure; the slip effect is suppressed, the chimerism between particles is enhanced, and the volume change is mainly manifested as the compaction effect.

The experimental results show that the confining pressure plays a decisive role in the volume change and deformation characteristics of rockfill materials. Under the condition of low confining pressure, the particle slip effect is dominant, resulting in significant lateral expansion. Under the condition of high confining pressure, the constraint between particles is enhanced, and the slip effect is weakened, thus inhibiting the lateral expansion and making the volume change mainly compaction.

As shown in Figure 8, the numerical simulation further reveals the formation and development of the shear plane of rockfill materials during axial loading. With the accumulation of axial strain, the cementation contact between particles is gradually destroyed. When the strain reaches the critical value, the shear surface presents a typical “X” type distribution. Under the condition of low confining pressure, the particle slip is more obvious, the shear surface characteristics are clear, and the obvious failure zone is formed along the “X” type distribution, which reflects the failure mechanism dominated by particle slip. Under the condition of high confining pressure, with the increase in confining pressure, the particle slip is limited, and the characteristics of the shear surface are gradually weakened, indicating that the high confining pressure effectively inhibits the formation of the shear surface and improves the shear strength of the rockfill. The higher confining pressure enhances the nesting and interlocking between the particles, which not only improves the overall stability of the rockfill, but also plays a positive role in the anti-sliding performance of the slope.

Under the same coarse grain content and confining pressure, the failure mode of numerical simulation is highly consistent with that of the indoor triaxial compression test. Whether it is the distribution of shear plane characteristics or the behavior of particle slip and contact failure, numerical simulation can effectively reveal the influence mechanism of confining pressure on the deformation of rockfill materials. This shows that PFC^2D^ numerical simulation can reliably reproduce the failure process of laboratory tests and reveal the mechanical response characteristics of rockfill materials during loading.

Furthermore, the PFC^2D^ model exhibits unique advantages in simulating particle sliding, shear band formation, and failure mechanisms. It enables detailed tracking of mesoscopic structural changes and mechanical behavior, providing a scientific foundation for investigating the mechanical properties and engineering stability of rockfill materials. Thus, PFC^2D^ serves as a valuable supplement to laboratory tests, verifying its reliability and effectiveness in studying soil deformation and failure mechanisms.

### 3.2. Analysis of Particle Breakage and Contact Force Chain Characteristics

**Figure 9:**
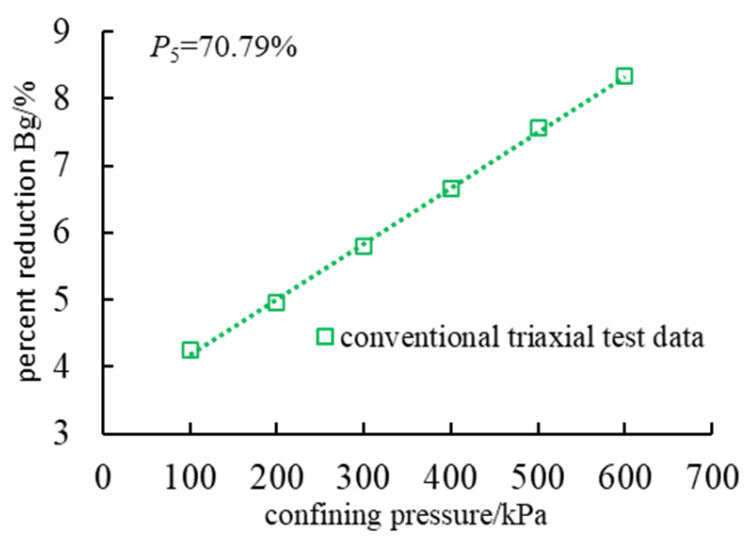
Relationship between particle breakage rate and confining pressure.

In the triaxial test, rockfill experienced different degrees of particle breakage. Using MARSAL crushing rate *B_g_* as a measure of the degree of fragmentation for the coarse grain content of 70.79% of the dam rockfill material [39], after the end of the test, the particle size distribution of each particle group was counted (Table 5), and the relationship curve between the crushing rate and the confining pressure was drawn (Figure 9).

It can be seen from Figure 9 that under the condition of constant gradation, the particle breakage rate increases significantly with the increase in confining pressure. Under low confining pressure conditions (100 kPa, 200 kPa), the particles are mainly slightly broken, and the slip and rotation between the particles play a dominant role. Under high confining pressure conditions (500 kPa, 600 kPa), the particle occlusion is enhanced, the number of contact points is increased, and the particles are more easily broken during the shear slip process, showing a significant confining pressure dependence. This trend is consistent with the research results of Sadeghian et al. [6] and Wang et al. [10]; that is, confining pressure has a significant effect on the breakage rate of rock fillers. However, Zhou et al.’s [15] study found that the crushing rate of some rock fillers is less sensitive to confining pressure, which may be related to the particle size distribution and particle characteristics of the material. In general, the increase in confining pressure promotes the interaction between particles, thus improving the degree of particle breakage and affecting the shear strength and stability.

**Figure 10:**
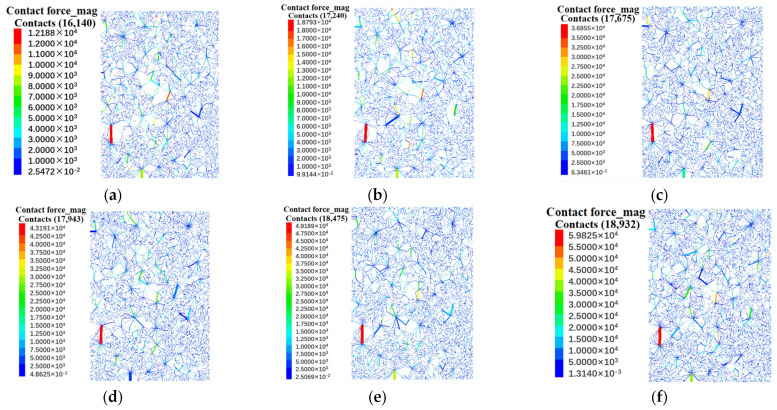
Distribution of internal force chain of rock fill under different confining pressure conditions: (**a**) σ_3_ = 100 kPa. (**b**) σ_3_ = 200 kPa. (**c**) σ_3_ = 300 kPa. (**d**) σ_3_ = 400 kPa. (**e**) σ_3_ = 500 kPa. (**f**) σ_3_ = 600 kPa.

Numerical simulation further reveals the influence of confining pressure on particle contact force and force chain network. Figure 10 shows the distribution of force chains after biaxial compression failure under different confining pressures. Table 6 records the number of force chains and the maximum contact force after shear failure.

**Figure 11:**
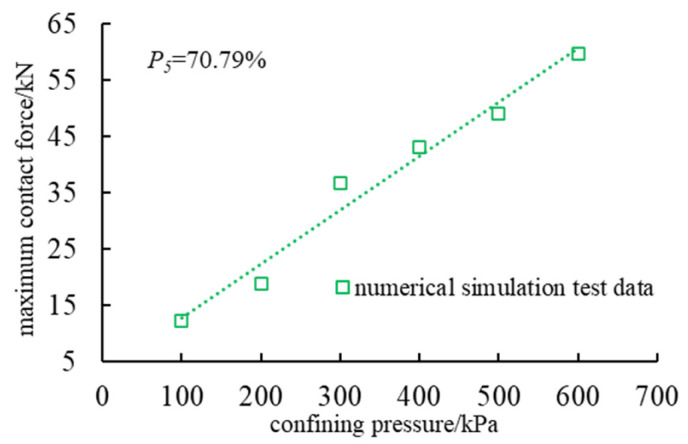
The relationship between particle contact stress and confining pressure.

The simulation results show that the maximum contact force between particles increases significantly with the increase in confining pressure, which is consistent with the change in shear strength measured by the laboratory test. At the same time, the number and contact times of strong and weak force chains increase with the increase in confining pressure, indicating that confining pressure has a significant effect on the stress state and failure mechanism of rockfill materials (Figure 11). Under the condition of high confining pressure, the relative slip of particles is limited, the contact force is enhanced, the occlusion is more significant, and a more complex and stable force chain network is formed.

In addition, with the increase in confining pressure, the main bearing direction of the force chain is gradually clear, and the stress transfer tends to be concentrated, forming a stable main chain structure. High confining pressure not only limits the free slip of particles but also promotes the evolution of the force chain network to an orderly distribution, which enhances the shear strength and overall stability. This result shows that the confining pressure dominates the stress transfer mechanism inside the rockfill and has a direct impact on the macroscopic strength characteristics.

Under the same coarse grain content and confining pressure, the failure modes of numerical simulation and the laboratory test are highly consistent. The variation trend of maximum contact force, shear failure mode, and force chain distribution characteristics shows good consistency, which further verifies the accuracy of PFC^2D^ in reliably reproducing the microscopic failure mechanism of the laboratory test. In addition, PFC^2D^ simulation can accurately capture the process of particle slip, shear surface formation, and failure, which provides a scientific basis for further study of the mechanical properties of rockfill materials. Therefore, the numerical model is not only an effective supplement to the laboratory test but can also be used to predict the long-term stability of rockfill under complex stress conditions, which provides an important reference for engineering applications.

## 4. Mesoscopic Motion of Particles and Crack Propagation Law

### 4.1. Numerical Simulation of Particle Motion Characteristics

**Figure 12:**
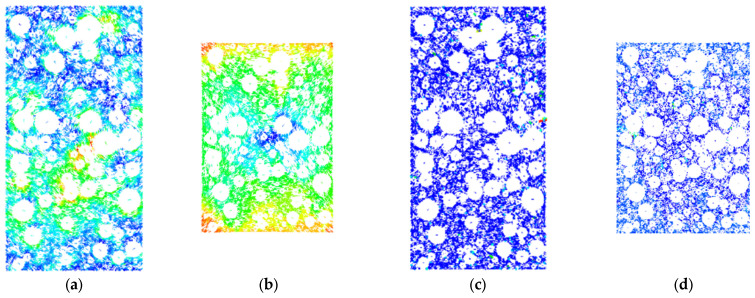
Motion characteristics of internal particles under confining pressure of 200 kPa: (**a**) Vector displacement field cloud diagram. (**b**) Vector displacement field cloud diagram. (**c**) Vector velocity field cloud picture. (**d**) Vector velocity field cloud picture.

**Figure 13:**
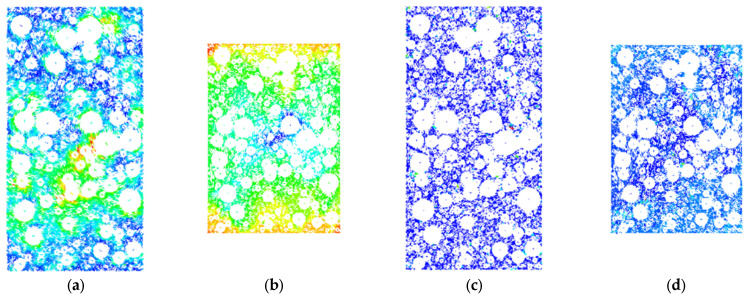
Motion characteristics of internal particles under confining pressure of 400 kPa: (**a**) Vector displacement field cloud diagram. (**b**) Vector displacement field cloud diagram. (**c**) Vector velocity field cloud picture. (**d**) Vector velocity field cloud picture.

**Figure 14:**
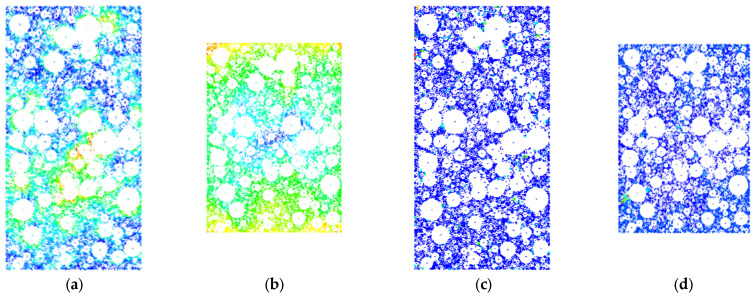
Motion characteristics of internal particles under confining pressure of 600 kPa: (**a**) Vector displacement field cloud diagram. (**b**) Vector displacement field cloud diagram. (**c**) Vector velocity field cloud picture. (**d**) Vector velocity field cloud picture.

In order to further analyze the movement characteristics of rockfill materials under the shear action of the indoor triaxial testing machine, and reveal its movement law, this paper uses PFC numerical simulation software to simulate the change process of rockfill materials under compression conditions. Taking the confining pressure of 200 kPa, 400 kPa, and 600 kPa as an example, after the shear failure occurs, the displacement vector cloud diagram and the instantaneous rate cloud diagram are derived. The specific results are shown in Figure 12, Figure 13 and Figure 14.

From Figure 12 to Figure 14, for rockfill materials with the same coarse grain content, under different confining pressure conditions, the motion state of rockfill particles before compression in the simulation experiment shows obvious pre-shear displacement, and the displacement direction is disordered. This irregular displacement vector and velocity vector reflect that the rockfill material is affected by confining pressure and self-weight before shearing, and the particles are squeezed and collided, in which the lower particles inhibit the movement of the upper particles. The color saturation in the displacement field (Figure 12a, Figure 13a and Figure 14a) represents the magnitude of particle displacement. Darker colors (towards red) indicate larger particle displacements, while lighter colors (towards blue) indicate smaller particle displacements. After the axial load is applied, the particle movement tends to be regular, and is manifested in the downward movement of the upper particles, the upward movement of the lower particles, and the movement of the side particles to both sides, which is consistent with the macroscopic performance in the laboratory test. In the velocity field (Figure 12b, Figure 13b and Figure 14b), the color saturation represents the particle movement speed. Darker colors indicate higher particle movement speed, while lighter colors indicate lower particle movement speed. From the perspective of micromechanics, the axial force promotes the displacement of the upper and lower particles along the direction of the force, while the side particles begin to move to both sides under the action of the axial force and the extrusion collision of the adjacent particles. The confining pressure failed to effectively limit the lateral displacement until the shear failure occurred, and the internal arrangement of rockfill particles changed regularly. In addition, the directions of the displacement vector and the velocity vector are not completely consistent, and some of them are even opposite, indicating that during the biaxial compression process, the extrusion and collision between particles may lead to changes in the spatial position of particles such as rolling and lifting. These phenomena reveal the complex interaction and motion mechanism of rockfill particles under stress.

### 4.2. Numerical Simulation of Particle Fracture Development Characteristics

In the PFC numerical simulation, the coarse and fine particles are cemented with each other through the parallel bond model to form a structurally stable whole. In the process of shear failure, with the increase in external shear force, the composition and geometric arrangement of particles change, resulting in the gradual weakening of cementation until cementation failure. This process will produce cracks between the rockfill particles, which will lead to the instability of the overall structure and ultimately affect the stability of the slope [40]. Therefore, it is of great significance to analyze the distribution characteristics of cracks in rockfill particles for the study of slope stability.

**Figure 15:**
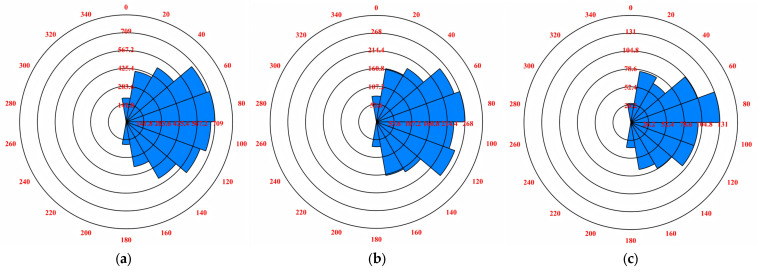

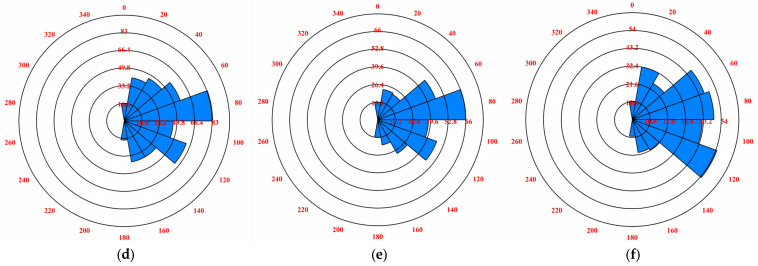
Rose diagram of shear fracture development of rockfill particles: (**a**) σ_3_ = 100 kPa. (**b**) σ_3_ = 200 kPa. (**c**) σ_3_ = 300 kPa. (**d**) σ_3_ = 400 kPa. (**e**) σ_3_ = 500 kPa. (**f**) σ_3_ = 600 kPa.

**Figure 16:**
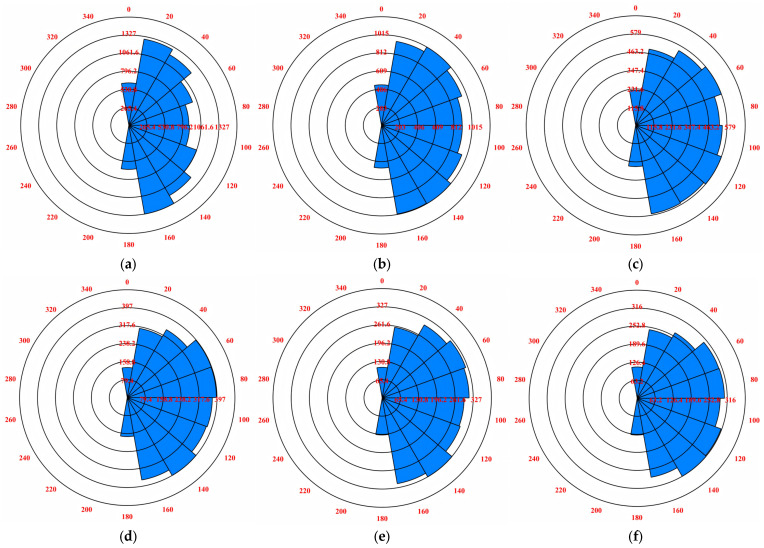
Rose diagram of tensile fracture development of rockfill particles: (**a**) σ_3_ = 100 kPa. (**b**) σ_3_ = 200 kPa. (**c**) σ_3_ = 300 kPa. (**d**) σ_3_ = 400 kPa. (**e**) σ_3_ = 500 kPa. (**f**) σ_3_ = 600 kPa.

The distribution characteristics of internal cracks in particles are important factors to characterize the damage degree of rockfill materials in the shear process. In order to analyze the fracture distribution characteristics of dam rockfill after biaxial compression failure, this paper uses the DFN Rosette program to derive the fracture rose diagram after shear failure, as shown in Figure 15 and Figure 16.

After biaxial compression simulation, Figure 15 and Figure 16 show that under the same coarse grain content, the development direction of shear cracks is concentrated from 60° to 120°, while the development direction of tensile cracks is concentrated from 20° to 160°. In general, under different confining pressure conditions, after the shear failure of rockfill materials, the development range of shear cracks and tensile cracks is roughly the same: both concentrated from 0° to 180°. This shows that the development range of cracks is not related to the structural characteristics between soil particles, and shows consistency. In addition, with the increase in confining pressure, the development degree of shear fissures decreases, indicating that high confining pressure helps to inhibit the development of shear fissures, thereby enhancing the stability of the soil.

At the same time, under the constant coarse grain content, there is a significant difference in the development degree of the shear cracks and tensile cracks of rockfill materials. Specifically, the development degree of tensile cracks is significantly higher than that of shear cracks. This is mainly because tensile cracks mainly occur between fine-grained samples. The presence of coarse particles increases the discontinuity between particles, which in turn, increases the linear density of internal tensile cracks and makes their development more significant.

## 5. Conclusions

In this paper, combined with an indoor triaxial compression test and PFC^2D^ numerical simulation, the macroscopic and mesoscopic mechanical properties of dam rockfill under different confining pressure conditions are systematically studied, and the following main conclusions are obtained:

(1) The experimental results indicate a significant positive correlation between the deviatoric stress and confining pressure in dam rockfill materials. As the confining pressure increases from 100 kPa to 600 kPa, the shear strength of the laboratory specimens increases from 769.43 kPa to 2140.98 kPa, while the shear strength of the numerical simulation specimens rises from 572.39 kPa to 2059.26 kPa. Under different confining pressures, rockfill materials exhibit distinct mechanical behaviors: pronounced dilative behavior under low confining pressure and contractive behavior under high confining pressure. Additionally, particle breakage intensifies with increasing confining pressure, confirming that confining pressure is a key factor influencing the mechanical behavior of rockfill materials.
(2) The weakening effect of particle breakage on the shear strength of rockfill is evident in both the experimental and numerical results, demonstrating that particle breakage significantly alters the grain composition and internal structure of rockfill. As the confining pressure increases, the breakage ratio rises from 4.25% to 8.33%, leading to a progressive reduction in shear strength. Particle breakage not only affects the macroscopic strength of rockfill but also significantly influences its mesoscopic mechanical response by altering the contact structure between particles. These changes ultimately result in a reduction in shear strength. Therefore, the combined effects of macroscopic strength degradation and mesoscopic contact structure evolution contribute to the overall mechanical response of rockfill materials.
(3) The characteristics of the shear band and force chain distribution are influenced by confining pressure. Numerical simulations reveal that the shear band in rockfill exhibits an X-shaped failure pattern, and as confining pressure increases, the development of shear fractures is inhibited, leading to improved overall stability. Meanwhile, the number of force chains increases from 16,140 under low confining pressure to 18,932 under high confining pressure, indicating more frequent and compact particle contacts. The maximum contact force between particles increases from 12.19 kN to 59.83 kN, suggesting that confining pressure enhances interlocking among particles, thereby improving shear strength and overall stability.
(4) The correlation between macroscopic and microscopic characteristics reveals that the mechanical behavior of rockfill under different confining pressures is deeply analyzed from the microscopic aspects of particle motion, crack distribution, and force chain characteristics. The research results reveal the influence mechanism of particle breakage, shear plane development, and force chain evolution on the macroscopic mechanical properties of rockfill materials, which provides an important reference direction for subsequent research.

## Figures and Tables

**Table 1:** Scale results of rockfill materials.

Rockfill	Percentage Content of Rockfill Particle Group/%						
	<2 mm	2–5 mm	5–10 mm	10–20 mm	20–40 mm	40–60 mm	60–100 mm
prototype gradation	14.59	14.64	17.52	11.48	14.86	13.40	13.51
scale gradation	14.59	14.64	21.65	14.19	18.37	16.56	-

**Table 2:** The main technical indexes of DJSZ-150 large coarse-grained soil dynamic and static triaxial testing.

Name	Parameter	Name	Parameter
Specimen size	Φ300 × 600 mm	Displacement measurement accuracy	0.3% F.S
Maximum axial static load	1500 kN	Pore water pressure	−0.1~3.0 MPa
Axial force resolution	0.1 kN	Back pressure	0~1.0 MPa
Surrounding pressure	0~3 MPa	Pressure measurement accuracy	±0.5% F.S
Displacement measurement range	0~300 mm	Axial loading rate (strain-type)	0.01~3.0 mm/min
Displacement resolution	0.001 mm	Hydraulic oil used	N46 anti-wear hydraulic oil

**Table 3:** Design of triaxial compression test scheme for rockfill materials.

Test Classification	Coarse Grain Content *P*_5_/%	Natural Moisture Content *w*/%	Density *ρ*/(g·cm^−3^)	Confining Pressure σ/kPa	
drainage by consolidation	70.79	3.45	2.32	100 kPa 300 kPa 500 kPa	200 kPa 400 kPa 600 kPa

**Table 4:** Summary of meso-parameter calibration results of dam rockfill materials.

Parameter	Numerical Value	Parameter	Numerical Value
coarse grain content *P*_5_*/*%	70.79	parallel bond cohesive force *c*/kPa	25.5
density *ρ*/(g/cm^3^)	2.32	angle of internal friction *φ*/◦	31
effective modulus *E*/kPa	5.0 × 10^8^	strain rate ε˙	0.1
stiffness ratio *k*	0.3	coefficient of restitution *e*	0.4
coefficient of friction *µ*	0.5	gravity g/(m/s^2^)	9.8

**Table 5:** Analysis results of rockfill particle breakage after triaxial test.

Test Classification	Coarse Grain Content *P*_5_/%	Confining Pressure σ/kPa	The Content of Each Particle Group with Different Particle Size (mm)/%						Percent Reduction
			<2	2~5	5~10	10~20	20~40	40~60	
conventional triaxial	70.79%	100	16.38	14.48	21.26	14.50	18.39	14.99	4.25
		200	16.61	14.41	21.20	14.55	18.46	14.77	4.95
		300	16.79	14.34	21.09	14.74	18.52	14.52	5.81
		400	17.25	14.29	21.12	14.85	18.20	14.29	6.65
		500	17.51	14.19	21.05	15.05	18.18	14.02	7.57
		600	17.85	14.13	21.01	15.09	18.13	13.79	8.33

**Table 6:** Internal force chain number and maximum contact force of rockfill under different confining pressures.

Coarse Grain Content *P*_5_/%	Confining Pressure σ/kPa	Number of Contact Force Chains N	Maximum Contact Force/kN	Shearing Strength/kPa
70.79	100	16,140	12.19	769.43
	200	17,240	18.79	1016.99
	300	17,675	36.86	1309.98
	400	17,943	43.19	1610.70
	500	18,475	49.19	1828.87
	600	18,932	59.83	2140.98

## Data Availability

The original contributions presented in this study are included in the article. Further inquiries can be directed to the corresponding authors.
